# Site-specific data on herbicide soil retention and ancillary environmental variables

**DOI:** 10.1016/j.dib.2019.104754

**Published:** 2019-11-02

**Authors:** Franca Giannini Kurina, Mónica Balzarini, Edgar Ariel Rampoldi, Susana Hang

**Affiliations:** a CONICET, Consejo Nacional de Investigaciones Científicas y Técnicas, Argentina; b Facultad de Ciencias Agropecuarias, Universidad Nacional de Córdoba, Argentina

**Keywords:** Adsorption coefficient, Glyphosate, Atrazine, Georeferenced data

## Abstract

This article presents original geospatial data on soil adsorption coefficient (Kd) for two widely used herbicides in agriculture, glyphosate and atrazine. Besides Kds, the dataset includes site-specific soil data: pH, total nitrogen, total organic carbon, Na, K, Ca, Mg, Zn, Mn, Cu, cation exchange capacity, percentage of sand, silt and clay, water holding capacity, aluminum and iron oxides, as well as climatic and topographic variables. The quantification of herbicides soil retention was made on a sample of soils selected by Conditionated Latin Hypercube method to capture the underlying edaphoclimatic variability in Cordoba, Argentina. The glyphosate data presented here has been used to evaluate statistical methods for model-based digital mapping (F. Giannini Kurina, S. Hang, R. Macchiavelli, M. Balzarini, 2019) [1]. The dataset is made publicly available to enable future analyzes on processes that leads the dynamics of both herbicides in soil.

**Table:** Specifications Table

Subject area	*Environmental Science.*
More specific subject area	*Non-point contamination in agriculture.*
Type of data	*Geographic Information System (GIS) database.*
How data was acquired	*Soil data were obtained from a spatial soil survey conducted in Córdoba Argentina* [2] *(355 sites). Glyphosate and atrazine Kd were determined (89 and 156 sites, respectively) in the soil laboratory at Facultad de Ciencias Agropecuarias, Universidad Nacional de Córdoba and imported to a geodatabase using Quantum GIS software.*
Data format	*Raw and spatialized*
Experimental factors	*Edaphoclimatic data were used to create the “Zone” factor* [9]
Experimental features	*Retention coefficients were spatialized using latitude, longitude and altitude information. All edaphoclimatic variables were projected to the same coordinated system Universal Transverse Mercator, Zone 20 South.*
Data source location	*Facultad de Ciencias Agropecuarias, Universidad Nacional de Córdoba* *Cordoba, Argentina*
Data accessibility	*Data are included with this data brief.*

**Table:** 

Value of the Data • Georeferenced herbicide soil adsorption coefficients, measured in an agricultural cropping area, could enhance knowledge about soil retention of potential contaminants in a regional scale. • The database is a valuable resource for investigators interested in geospatial research and contamination process as well for policy makers. • The additional value of this data is to support methodological researches on spatial multivariate analysis

## 1 Data

**Fig. 1:**
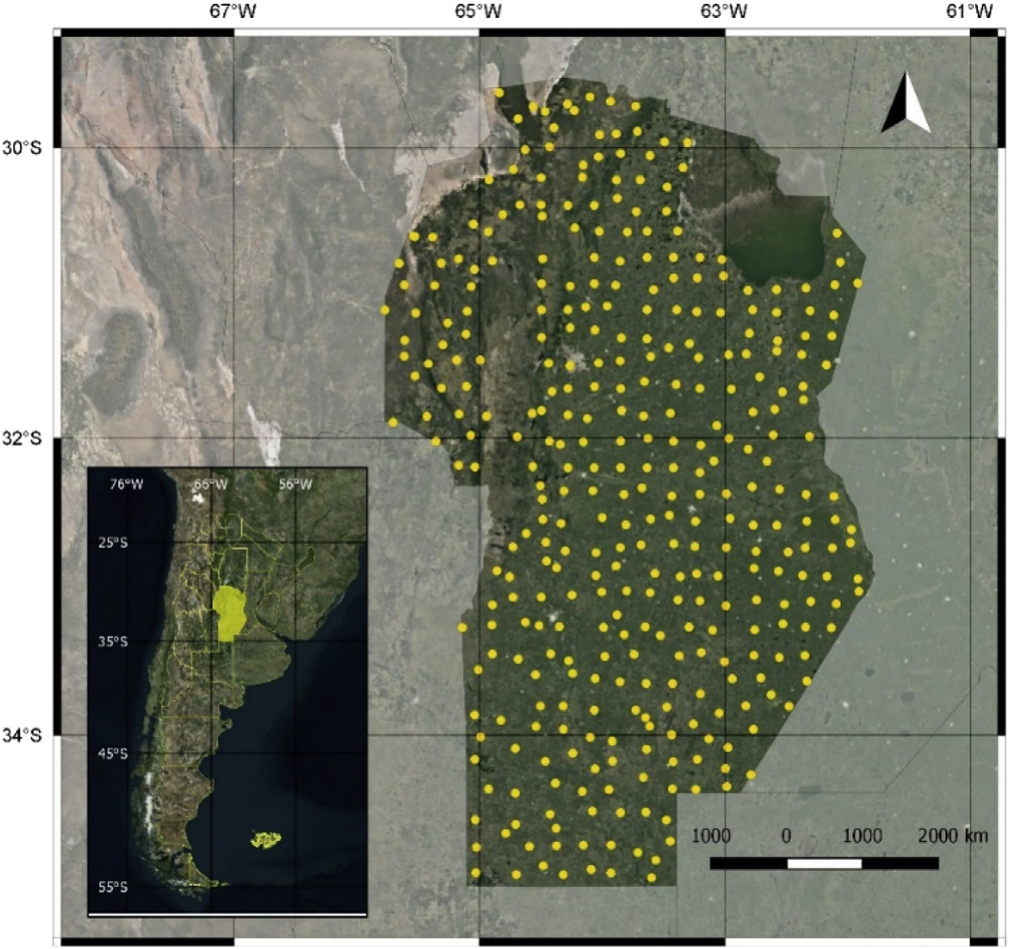
Cordoba, Argentina (29°–35°S, and 61° to 65°W). Sample sites.

Data have been cataloged for Kd of glyphosate (n = 89 sites) and atrazine (n = 156) found in soil. The Kd coefficient parametrizes the herbicide retention process. It expresses the relationship between the concentrations of the agrochemical between the solid phase and the solution of soil. Data also included environmental variables (edaphic, topographic and climatic) for a total of 355 geo-referenced sites (Fig. 1), from which soil sample were collected. Measured soil variables were pH, Total Nitrogen, Soil Organic Carbon, Na, K, Ca, Mg, Zn, Mn, Cu, Cation Exchange Capacity, percentage of Sand, Silt and Clay, water holding capacity, and aluminum and iron oxides [2]. Topographic (Elevation) and climatic data (annual cumulative precipitation and mean annual air temperature) were extracted from open global databases [3,4]. Attributes of the database are described in Table 1. The dataset table is provided as an Excel file (Microsoft Corporation, Redmond, Washington) and as an interactive map KML file (Keyhole Markup Language) in the supplementary material. The glyphosate data presented here has been used to evaluate statistical methods for model-based digital mapping (F. Giannini Kurina, S. Hang, R. Macchiavelli, M. Balzarini, 2019) [1].

**Table 1:** Herbicides adsorption coefficients and environmental variables in spatialized sites. Cordoba, Argentina.

Variable	Units	Description
ID_2	–	Identification code
X UTM20	m	Universal transverse mercator, zone 20 South coordinates reference system
Y UTM20	m	Universal transverse mercator, zone 20 South coordinates reference system
pH	–	pH in water 1:2.5 (soil:water)
EC	dS m^−1^	Electrical conductivity in water 1:2.5 (soil:water)
SOC	g kg^−1^	Soil organic carbon by 1 N K_2_Cr_2_O_7_ wet combustion, Walkley and Black method [5]
TN	% p:p	Total Nitrogen, Kjeldahl method [5]
Mn	mg kg^−1^	Extractable Manganese, extraction by Mehlic-3 [6]
Cu	mg kg^−1^	Extractable Copper, extraction by Mehlic-3 [6]
Zn	mg kg^−1^	Extractable Zinc, extraction by Mehlic-3 [6]
WHC	%	Water holding capacity, 300 kPa with a pressure cooker [7]
Silt	%	Sand content, Robinson pipette method [5]
Lime	%	Lime content, Robinson pipette method [5]
Clay	%	Clay content, Robinson pipette method [5]
Al(Ox)	%	Aluminum oxides [8]
Fe(Ox)	%	Iron oxides [8]
P	ppm	Phosphorus extractable, extraction by the Bray and Kurtz 1, colorimetric [5]
K	Ppm	Exchangeable Potassium (ppm), flame Photometry [5]
Ca	ppm	Exchangeable Calcium (ppm), complexometric [5]
Na	ppm	Exchangeable Sodium (ppm), flame Photometry [5]
Mg	ppm	Exchangeable Magness (ppm), complexometric [5]
CEC	Cmol kg^−1^	Cation exchange capacity [5]
Elevation	m.a.s.l	Elevation, Digital Elevation Model STRM [3]
Tm	°C	Mean air annual temperature, BIOCLIM [4]
pp	mm	Annual cumulated precipitations, BIOCLIM [3]
TvsPP	°C mm^−1^	Tm over pp
Kdg	Lkg^−1^	Glyphosate adsorption coefficient
Kda	Lkg^−1^	Atrazine adsorption coefficient
Kdg_measured	–	Sites with glyphosate Kd measured
Kda_measured	–	Sites with atrazine Kd measured
Zone (4)	–	Edaphoclimatic zoning [9]

## 2 Experimental design, materials and methods

Soil samples were taken from the upper 15 cm of soil in a regular 40 × 40 km grid (Fig. 1). Soil properties were measured according with the methods listed in Table 1. Topographic variables were obtained from the Digital Elevation Model provided by the STRM (Shuttle Radar Topography Mission [3]) and climatic information, taken from the global database of climatic analysis (BIOCLIM [4]). Using Conditioned Latin Hypercube [10] method a sample of 89 sites was obtained to determine glyphosate retention and another sample 159 sites to quantify atrazine retention. For both herbicides, the Kd coefficient were determined in each soil sample according to the batch-equilibrium technique for the preparation of soil suspensions. For fortifications, the standards had a >98% of purity and were provided by Sigma-Aldrich standards. A 2g soil mass was put in 50 ml centrifuge tubes where 10 ml of the fortification solution (concentrations of 10 mgL-1 for glyphosate and 20 mgL-1 for atrazine) were added. The fortified soils were first taken to a shaker for 24 h at 25 ± 1 °C, then centrifuged 5 min at 4000 rpm. Finally, the remaining supernatant was filtered by 0.45μm cellulose filters to a 1.5ml autosampler vials. The equilibrium concentration of each herbicide (Ceq) was quantified by high-pressure liquid chromatography (HPLC). For atrazine, a Photodiode Array Detectors (PDA) in a stationary phase octadecylsilane (C18) and for glyphosate post-column derivatization and fluorometric detection. The adsorbed concentration (Cad) was calculated as the difference between the initial concentration and the concentration at equilibrium in the solution. Finally, Kd was calculated as the following ratio Cad/Ceq. Variables in the built database are presented in Table 1.
